# Impact of dietary *Alpinia Katsumadai* extracts on production performance, meat quality, and gene expression in AMPK signaling regulatory pathway of Wuzhishan pigs

**DOI:** 10.3389/fvets.2025.1563498

**Published:** 2025-06-05

**Authors:** Hongzhi Wu, Xilong Yu, Xiaoyu Zhang, Euphrème Ipemba, Ghislain Boungou Bakala, Lessebe Gambou Dieu Leveut, Weiqi Peng, Fengjie Ji, Hanfeng Li, Ting Cao, Renlong Lv

**Affiliations:** ^1^Tropical Crops Genetic Resources Research Institute, Chinese Academy of Tropical Agricultural Sciences, Haikou, China; ^2^National Centre for Crop Disease Control, Ministry of Agriculture, Animal Husbandry and Fisheries, Brazzaville, Republic of Congo; ^3^China-Aid-Congo (B) Agricultural Technology Demonstration Center, Brazzaville, Republic of Congo

**Keywords:** *Alpinia Katsumadai* extracts, meat quality, amino acids, long-chain fatty acids, AMPK signaling pathway

## Abstract

Pork is one of the most consumed meats globally, especially in China, Europe, and North America. Meat quality is a multifaceted concept that can be assessed from several key perspectives, such as palatability, nutritional value, and processing characteristics. This study investigated the effects of dietary *Alpinia Katsumadai* extracts on meat quality, amino acids, and long-chain fatty acids profiles, as well as gene expressions in the AMPK signaling regulatory pathway of Wuzhishan pigs. Forty-eight pigs were selected and randomly divided into four groups, with six replicates and two pigs for each replicate. The control group (CON) was administered a basal diet, while the experimental groups received basal diets supplemented with 400, 600, and 800 mg/kg of *Alpinia Katsumadai* extracts, respectively (designated as AK1, AK2, and AK3). The results showed that compared with the control group, the components of the treated groups exhibited significant differences. Specifically, the muscle inosinic acid, intramuscular fat, and triacylglycerol contents were higher (*p* < 0.05). In terms of quality, the b* and L* values of the longissimus dorsi muscle at 45 min post-slaughter were higher (*p* < 0.05), and drip loss was lower (*p* < 0.05). Regarding metabolism, the muscle C14:0, C16:0, C18:3 n3, C18:2 n6c, C20:4 n6, and PUFA proportions were higher (*p* < 0.05), while MUFA proportions were lower (*p* < 0.05). Additionally, the relative expressions of *ACC*, *PRKAA1*, *PRKAG1*, and *MyHC I* in the muscle were higher (*p* < 0.05), whereas those of *PRKAB1*, *PRKAB2*, *PPARγ*, and *MyHC IIb* were lower (*p* < 0.05). In the liver, the relative expressions of *ACC*, *PRKAA1*, and *PRKAG1* were higher (*p* < 0.05), and those of *PPARγ*, *PRKAB1*, *PRKAB2*, and *PGC-1α* were lower (*p* < 0.05). These findings indicate that *Alpinia Katsumadai* extracts enhance the meat composition, quality, amino acids, long-chain fatty acids, and gene expressions in the AMPK signaling regulatory pathway of Wuzhishan pigs. The optimal dosage identified in this study is 600 mg/kg.

## 1 Introduction

Pork is among the most consumed meats globally, especially in China, Europe, and North America (1, 2). Pork quality represents a multifaceted construct encompassing sensory attributes, technical parameters, and nutritional composition (3). Meat quality is a multidimensional concept that can be assessed from several key perspectives, such as palatability, nutritional value, and processing characteristics (4, 5). Fatty acid and amino acid compositions directly and indirectly impact meat quality, including sensory characteristics, nutritional and health values, and other aspects (6). Adenosine 5′-monophosphate (AMP)-activated protein kinase (AMPK) signaling pathway is a key energy sensor and metabolic regulator within the cell, playing an essential role in cellular energy balance and metabolic regulation (6, 7). The AMPK negatively regulates adenosine triphosphate (ATP)-consuming biosynthetic processes, including gluconeogenesis, lipid, and protein synthesis (8, 9). In animal meat production, activating the AMPK can promote the generation of ATP and inhibit the consumption of ATP, thereby regulating the energy balance within the muscle (9). By controlling the activity of the AMPK, the rate and extent of glycolysis can be altered, affecting the growth and maturation of muscle tissue and meat quality (10).

Plant extracts play a multifaceted role in enhancing meat quality (11, 12). They can improve the sensory characteristics and nutritional value of meat products while bolstering their safety and health benefits, thereby contributing positively to producing high-quality meat products (13). *Alpinia Katsumadai* extracts, derived from the dried seeds of the nearly ripe *Alpinia Katsumadai* fruit, contain various bioactive components. These include volatile oils like eucalyptol, *α*-humulene, trans-farnesol, and flavonoid compounds like cardamomin, alpinetin, and saponins (14, 15). These functional components may contribute to the regulation of meat quality. Essential oils can enhance the sensory characteristics of meat by improving its flavor and tenderness (16). Flavonoids, known for their antioxidant properties, may help maintain the freshness and nutritional value of meat (17). There is little research on *Alpinia Katsumadai* extracts regarding meat quality, and most of it focuses on preserving meat. Pogačar *et al.* reported that *Alpinia Katsumadai* extracts could be used in pharmaceutical and food products (18). Klančnik *et al.* found *Alpinia Katsumadai* extracts reduced microbiological risk in minced meat (19).

The Wuzhishan pig is a small-sized pig native to Wuzhishan City in Hainan Province, China. It is characterized by suboptimal growth performance and slow growth rates. However, its meat quality is remarkably superior, with a high lean meat content and substantial intramuscular fat (20). The meat is flavorful, tender, succulent, and rich in amino acids and unsaturated fatty acids, rendering it both nutritious and healthful (21). This study sought to explore the possible effects of *Alpinia Katsumadai* extracts on the meat quality of Wuzhishan pigs through the assessment of various indicators, such as intramuscular fat content, meat tenderness, and the amino acids and fatty acids composition. It also aimed to preliminarily assess the viability of incorporating *Alpinia Katsumadai* extracts into pig production practices. Moreover, the research attempted to lay the groundwork for the potential use of these extracts by investigating their possible impact on gene expression within the AMPK signaling regulatory pathway, which may subsequently influence meat quality.

## 2 Materials and methods

### 2.1 Experimental material

Wuzhishan pigs (sows or castrated boars), second parity, with an average body weight of 25.00 ± 1.00 kg, the same genetic background, and health status, were purchased from the Wuzhishan Pig Breeding Base in Danzhou, Hainan Province.

*Alpinia Katsumadai* extracts were purchased from Shaanxi Baichuan Biotechnology Co., Ltd., Xian, China. The active ingredients of *Alpinia Katsumadai* were analyzed using liquid chromatography and mass spectrometry at the Pony Testing International Group Company in Beijing, China. The essential oil content in the *Alpinia Katsumadai* extracts was 1.26%, with terpenes comprising 92.70% of this total. The major terpenes identified included 1,8-cineole (19.18%), pinene (11.76%), terpinen-4-ol (10.42%), thujone (10.01%), and p-cymene (9.28%).

Triglyceride assay kits were purchased from Shanghai Sangon Biotechnology Co., Ltd., Shanghai, China.

### 2.2 Experiment design

In this study, the *Alpinia Katsumadai* extract is the single influencing factor. *Forty-eight* Wuzhishan pigs were randomly allocated into four groups, with six replicates and two pigs per replicate. The basal diets for pigs were corn-soybean meal-type diets formulated according to the NCR (1998; 2012) standards, and the composition of the basal experimental diets and nutritional levels are shown in Table 1. The control group (CON) was administered a basal diet, while the experimental groups received basal diets supplemented with 400, 600, and 800 mg/kg of *Alpinia Katsumadai* extract, respectively (designated as AK1, AK2, and AK3), in powder form, according to recommended usage on the product instructions. The experiment period was 90 days. The pig house was maintained at 28–30°C and 60–70% humidity. Lighting was provided for 16 h daily. Fecal waste was cleared twice daily, and the house was disinfected weekly with a 0.10% povidone-iodine solution to ensure hygiene.

**Table 1:** Composition (kg/100 kg) of the basal experimental diets^a^ for Wuzhishan pigs.

Ingredients	Contents	Nutrient levels, on an air-dry basis	Contents
Corn, %	33.80	Digestible energy^c^, DE, MJ/kg	13.61
Soybean meal, %	14.40	Crude protein^d^, CP, %	17.50
Wheat, %	9.00	Calcium^d^, Ca, %	0.85
Low protein whey powder, %	5.00	Total Phosphorus^d^, P, %	0.75
Fish meal, %	3.00	Available phosphorus^d^, AP, %	0.53
Corn gluten meal, %	10.00	Lysine^d^, Lys, %	1.25
Wheat bran, %	3.00	Methionine^d^, Met, %	0.35
Soybean oil, %	5.00	Threonine^d^, Thr, %	0.78
Mountain flour, %	0.80	Tryptophan^d^, Try, %	0.29
Calcium hydrogen phosphate, %	2.00		
Salt, %	0.30		
*L*-Lysine hydrochloride, %	0.60		
Threonine, Thr, %	0.10		
Tryptophan, Try, %	0.10		
Saccharose, %	2.50		
Glucose, %	2.50		
Premix^b^, %	2.00		
Feed mold inhibitor, %	1.00		
Sweetening agent, %	0.30		
Zeolite powder, %	4.60		

a Based on the NRC (1998; 2012) nutrient requirements for pigs.

b The premix provided the following per kg of diet: VA 2,000 IU, VD 200 IU, VE 45.00 IU, VK 0.50 mg, VB_1_ 1.00 mg, pantothenic acid 12.00 mg, nicotinic acid 10.25 mg, VB_6_ 3.85 mg, VB_12_ 15.00 ug, folic acid 1.35 mg, biotin 0.21 mg, VC 200 mg, Mn as manganese sulfate 20.00 mg, Fe as ferrous sulfate 80.00 mg, Cu as copper sulfate 5.00 mg, I as potassium iodide 0.14 mg, Se as sodium selenite 0.15 mg.

c Calculated value (NRC, 1998; 2012).

d Analyzed content.

### 2.3 Sample collection and indicator testing

At the conclusion of the experiment, the pigs were subjected to a 12 h fasting period, during which they were permitted free access to water. We chose one pig close to the average weight for slaughter from each replicate.

#### 2.3.1 Production performance

The initial and final body weights of each pig were measured at the start and end of the experiment, respectively, to calculate the average initial and final body weights. Following slaughter, the head, hooves, tail, and viscera, excluding the kidneys and perirenal fat, were removed. The remaining two halves of the carcass were weighed separately, and the sum of these weights was recorded as the total carcass weight.

#### 2.3.2 pH value and meat color

Following slaughter, approximately 200 grams of the longissimus dorsi muscle should be placed into a self-sealing bag and stored in a refrigerator maintained at 4°C. At 45 min and 24 h post-slaughter, a portable pH meter (model PHS-3E, manufactured in Shanghai, China) should be utilized to randomly select two distinct locations on the muscle for pH value measurement. In addition, a colorimeter (model Konica Minolta CM-700d, from Japan) should be employed to randomly choose two positions to assess the redness (a*), yellowness (b*), and lightness (L*) values of the muscle.

#### 2.3.3 Shear force and drip loss

Following slaughter, a 50.00 g portion of the longissimus dorsi muscle was carefully excised from a consistent anatomical region, with excess fat and fascia meticulously removed. The muscle tissue was then sectioned into uniform cubic samples, each measuring 2.54 cm × 2.54 cm × 2.54 cm and weighing approximately 10.00 g. These samples were used for two distinct analyses.

For the shear force analysis, the samples were individually placed into cooking bags, to which an appropriate volume of water was added. The bags were sealed and immersed in a water bath at approximately 70°C. The samples were cooked for about 30 min, ensuring that the meat was fully cooked yet not overcooked. Upon completion of the cooking process, the samples were removed from the bags and allowed to cool to room temperature. The shear force of the samples was then assessed using a texture analyzer (model CLM-3B, Harbin, China).

For the drip loss analysis, the initial mass of each sample was precisely determined using an analytical balance (FA2204, Shanghai, China), and the data were meticulously recorded. The samples were subsequently suspended by a string, ensuring adequate spacing to prevent contact between them, and placed in a well-ventilated environment at 4°C. After a 45 min and 24 h hanging period, the samples were retrieved, their surface moisture gently blotted with absorbent paper, and their mass was re-measured using the analytical balance. The drip loss rate was calculated based on the initial and post-hanging mass data.

#### 2.3.4 Intramuscular fat and triacylglycerol

Following slaughter, a 100 g sample was taken from the longissimus dorsi muscle, ensuring the same anatomical location was used for consistency. Excess fat and fascia were carefully removed, and the muscle tissue was cut into small pieces. These pieces were then placed in an oven set at approximately 65°C until they reached a constant weight, indicating complete drying. The dried muscle pieces were subsequently ground into a fine powder. An accurate 5.00 g sample of this powder was weighed and placed into a filter paper sleeve suitable for a Soxhlet extractor (model BSXT-06, Shanghai, China). The sleeve was inserted into the Soxhlet apparatus, and an appropriate volume of anhydrous ether was added as the solvent. The extraction process involved heating the apparatus in a water bath for 8 h to ensure complete extraction and evaporation of the ether. After extraction, the filter paper sleeve containing the residue was placed back into the oven at around 105°C for 2 h to dry thoroughly. Once cooled, the sample was weighed again to determine the intramuscular fat content through calculation.

An additional 5.00 g sample of the longissimus dorsi muscle was weighed and placed into a 15 mL centrifuge tube for further analysis. An appropriate volume of physiological saline was added, and the mixture was homogenized using a tissue homogenizer (model Tissuelyser-192, Shanghai, China). The homogenate was then centrifuged at 3000 rpm for 15 min. The supernatant was carefully transferred to a 2 mL Eppendorf tube for storage and subsequent testing, following the specific instructions for the assay.

#### 2.3.5 Inosinic acid and amino acids

Following slaughter, a 200 g sample of fresh porcine longissimus dorsi muscle was excised, with non-muscular tissues such as fat and fascia meticulously removed. The muscle tissue was minced and ground using a pulverizer (JY-100, Shanghai, China). The ground samples were stored at −80°C for subsequent analysis. For sample preparation, 10.00 g of the ground muscle tissue (measured to an accuracy of 0.01 g) was precisely weighed and transferred into a 50 mL centrifuge tube. A 25.00 mL volume of 5.00% perchloric acid solution was added, and the mixture was homogenized. The homogenized sample was then subjected to ultrasonic extraction for 30 min, followed by centrifugation at 10,000 revolutions per minute for 10 min. The resulting supernatant was carefully decanted and filtered through medium-speed qualitative filter paper. The filtrate was collected and its pH adjusted to approximately 6.5 using a 5.00 mol/L sodium hydroxide solution. Subsequently, the filtrate was passed through a 0.45 μm microporous filter membrane to yield the final sample solution for analysis.

The analytical procedure involved using a COSMOSIL Packed Column C18 (4.60 mm × 250 mm, 5 μm) maintained at a column temperature of 40°C. The mobile phase consisted of a 0.05 mol/L potassium dihydrogen phosphate solution, with the pH adjusted to 5.40 using 8 g/100 mL of dipotassium hydrogen phosphate. The elution was performed isocratically at a flow rate of 0.80 mL/min. Detection was carried out at a wavelength of 254 nm, with an injection volume of 10.00 μL.

#### 2.3.6 Long-chain fatty acids

Following slaughter, a 100 g sample from the same section of the longissimus dorsi muscle was obtained, and the fascia was removed. The sample was then ground using a meat grinder and placed in an oven to dry at 65°C until a constant weight was achieved. The dried meat sample was subsequently ground with a food pulverizer and stored in a self-sealing plastic bag at room temperature for later use. A 0.50 g portion of the dried and ground meat sample was weighed and placed in a 15 mL centrifuge tube. 4.00 mL of isooctane was added, and the mixture was thoroughly combined for 30 s using a vortex mixer. The tube was then placed in a constant temperature shaker set at 37°C and left to shake overnight. After this, 4.00 mL of a 2.00 mol/L potassium hydroxide-methanol solution was added, thoroughly combined for 30 s using a vortex mixer, and rapid methylation was performed. The mixture was left to stand for 30 min to allow the layers to separate, after which approximately 1.00 g of sodium bisulfate was added to the test tube and vigorously shaken to neutralize the remaining potassium hydroxide until the solution became transparent. The supernatant, following methylation, was filtered through a 0.45 μm microporous filter membrane in preparation for testing.

Gas chromatography detection conditions were as follows: Column: Polyethylene glycol intense polar stationary phase, with a column length of 30 m, an inner diameter of 0.250 mm, and a film thickness of 0.25 μm. Detector: Flame ionization detector (FID). Injector temperature: 220°C. Detector temperature: 280°C. Programmed temperature rise: 50°C held for 1 min, then increased at 25°C/min to 220°C, held for 0 min, then increased at 3°C/min to 230°C, held for 18 min, and run for a total of 35 min. Carrier gas: Nitrogen. Gas flow rates: Nitrogen,30 mL/min; Hydrogen, 40 mL/min; Air, 450 mL/min. Split ratio: 50:1. Injection volume: 1 μL.

#### 2.3.7 RNA extraction and quantitative analysis of mRNA with RT qPCR

Total RNA was extracted from approximately 1.50–2.00 g of longissimus dorsi muscle and liver tissue (each roughly the size of a soybean) using an animal tissue RNA extraction kit, following the manufacturer’s protocol. RNA quality and integrity were assessed by 2% agarose gel electrophoresis. RNA concentration and purity were measured using a NanoPhotometer^®^ spectrophotometer (Implen, Germany), and RNA samples with an A260/A280 ratio between 1.8 and 2.1 were used for downstream applications. The primer sequences used in this study are listed in Table 2.

**Table 2:** Primers used for quantitative RT-qPCR.

Genes	Name	Primer sequences (5′-3′)	Product length, bp	Accession No.
*ACC*	*Acetyl-CoA carboxylase*	F: GGCCATCAAGGACTTCAACC	120	NM_001114269
		R: ACGATGTAAGCGCCGAACTT		
*PPARγ*	*Peroxisome proliferator-activated receptor gamma*	F: GAGGGCGATCTTGACAGGAA	124	NM_214379
		R: GCCACCTCTTTGCTCTGCTC		
*PRKAA1*	*Protein kinase AMP-activated catalytic subunit alpha 1*	F: GGCTCAGTTAGCAACTATTCG	120	NM_001167633
		R: GTCAACAGGAGAAGAGTCAAG		
*PRKAB1*	*Protein kinase AMP-activated non-catalytic subunit beta 1*	F: GGACACAGGCATTTCTTG	100	NM_001243621
		R: CACCATCACTCCATCCTT		
*PRKAB2*	*Protein kinase AMP-activated non-catalytic subunit beta 2*	F: GTCTGAAGGAGGCAAGGA	108	NM_001243683
		R: AAGGTCTAGGATGGCAACA		
*PRKAG1*	*Protein kinase AMP-activated non-catalytic subunit gamma 1*	F: GCATCCTCAAGTTCCTCAA	106	NM_001001642
		R: ATAGCAATGTTGGCATAGGT		
*PGC-1α*	*Peroxisome proliferator-activated receptor-gamma coactivator 1-alpha*	F: TGACAGCGAAGATGAAAGTGA	132	XM_021100444.1
		R: GATTTGGGTGGTGATACGG		
*FAS*	*Fatty acid synthase*	F: ACACCTTCGTGCTGGCCTAC	112	NM_001099930
		R: ATGTCGGTGAACTGCTGCAC		
*LPL*	*Lipoprotein lipase*	F: ATCTGCGGGATACACCAAGC	110	NM_214286
		R: CCAAGGCTGTATCCCAGGAG		
*SREBP-1c*	*Sterol regulatory element binding protein-1c*	F: GCGACGGTGCCTCTGGTAGT	96	NM_214157.1
		R: CGCAAGACGGCGGATTTA		
*MyHC I*	*Myosin heavy chain I*	F: AAGGGCTTGAACGAGGAGTAGA	137	AB053226
		R: TTATTCTGCTTCCTCCAAAGGG		
*MyHC IIb*	*Myosin heavy chain IIb*	F: ATGAAGAGGAACCACATTA	166	AB025261
		R: TTATTGCCTCAGTAGCTTG		
*β-actin*	*–*	F: CCTGCGGCATCCACGAAAC	123	XM-003124280.3
		R: TGTCGGCGATGCCTGGGTA		
*GAPDH*	*Glyceraldehyde-3-phosphate dehydrogenase*	F: TCGGAGTGAACGGATTTGGC	95	NM-001206359.1
		R: GAAGGGGTCATTGATGGCGA		

Complementary DNA (cDNA) was synthesized using the PrimeScript^®^ RT reagent Kit with gDNA Eraser (TaKaRa, Dalian, China). The gDNA removal reaction was prepared by mixing 2 μL of 5 × gDNA Eraser Buffer, 1 μL of gDNA Eraser, and 1 μg of total RNA, and adjusting the volume to 10 μL with RNase-free water. Reverse transcription was conducted in a 20 μL system consisting of the above 10 μL gDNA-treated RNA, 1 μL of RT Primer Mix, 4 μL of 5 × PrimeScript Buffer, 1 μL of PrimeScript RT Enzyme Mix I, and 4 μL of RNase-free water. The thermal conditions for cDNA synthesis were 37°C for 15 min and 85°C for 5 s, followed by storage at −20°C until use.

Quantitative real-time PCR was performed using the Applied Biosystems PRISM 7500 Fast Real-Time PCR System (Foster City, CA, United States). Each 20 μL PCR reaction contained SYBR^®^ Premix Ex Taq II, 10 ng of cDNA template, and 0.4 μM each of forward and reverse primers (synthesized by Sangon Biotech, Shanghai, China). The thermal cycling conditions consisted of an initial denaturation at 95°C for 30 s, followed by 40 cycles of denaturation at 95°C for 5 s and annealing/extension at 60°C for 34 s. Following amplification, a melt curve analysis was performed from 60°C to 95°C, with 0.3°C increments every 15 s, to verify the specificity of amplification and the absence of primer dimers or nonspecific products. *Glyceraldehyde-3-phosphate dehydrogenase* (*GAPDH*) and *β-actin* were used as internal controls. The relative mRNA levels of ACC (Acetyl-CoA carboxylase), *PPARγ* (*Peroxisome proliferator-activated receptor gamma*), *PRKAA1* (*Protein kinase AMP-activated catalytic subunit alpha 1*), *PRKAB1* (*Protein kinase AMP-activated non-catalytic subunit beta 1*), *PRKAB2* (*Protein kinase AMP-activated non-catalytic subunit beta 2*), *PRKAG1* (*Protein kinase AMP-activated non-catalytic subunit gamma 1*), *PGC-1α* (*Peroxisome proliferator-activated receptor-gamma coactivator 1-alpha*), *FAS* (*Fatty acid synthase*), *LPL* (*Lipoprotein lipase*), *SREBP-1c* (*Sterol regulatory element binding protein-1c*), *MyHC I* (*Myosin heavy chain I*), and *MyHC IIb* (*Myosin heavy chain IIb*) were determined using the 2^−ΔΔCt^ method (22) and normalized to the expression levels of *GAPDH* and *β-actin*. The final gene expression results were calculated based on the average expression levels of the two internal controls.

### 2.4 Statistical analysis

Statistical analyses were conducted using SPSS Statistics software (version 20.0, International Business Machines Corporation, Armonk, NY, United States). Data were expressed as mean ± SEM. Different treatments were statistically compared using one-way ANOVA or Welch ANOVA after the Kolmogorov–Smirnov and variance homogeneity test. Statistical differences among groups were assessed using Duncan’s multiple range test. The test results of all analyses were considered significant at *p* < 0.05.

## 3 Results

### 3.1 Effects of *Alpinia Katsumadai* extracts on the production performance of Wuzhishan pigs

There were no significant differences (*p* > 0.05) in initial body weight, final body weight, carcass weight among the groups (Table 3).

**Table 3:** Effects of *Alpinia Katsumadai* extracts on the production performance of Wuzhishan pigs.

Items	CON	AK1	AK2	AK3	*p*-value
Initial body weight, kg	25.05 ± 0.91	24.95 ± 1.00	25.00 ± 0.95	24.96 ± 0.98	0.9367
Final body weight, kg	33.56 ± 0.62	33.64 ± 0.58	33.63 ± 0.69	33.61 ± 0.75	0.8296
Carcass weight, kg	22.45 ± 0.65	22.53 ± 0.92	22.51 ± 0.84	22.52 ± 0.96	0.7963

### 3.2 Effects of *Alpinia Katsumadai* extracts on the longissimus dorsi muscle composition of Wuzhishan pigs

**Figure 1:**
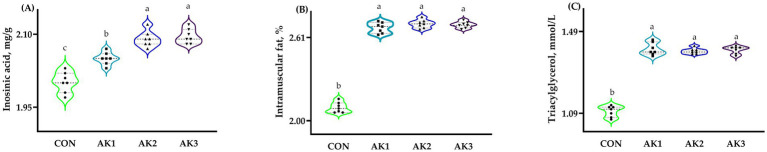
Effects of *Alpinia Katsumadai* extracts on the longissimus dorsi muscle composition of Wuzhishan pigs. **(A)** The inosinic acid contents, mg/g, in CON, AK1, AK2, and AK3 were 2.00 ± 0.01^c^, 2.05 ± 0.01^b^, 2.09 ± 0.02^a^, 2.10 ± 0.01^a^, respectively, *p* = 0.0137; **(B)** The intramuscular fat contents, %, in CON, AK1, AK2, and AK3 were 2.10 ± 0.12^b^, 2.68 ± 0.20^a^, 2.72 ± 0.11^a^, 2.71 ± 0.15^a^, respectively, *p* = 0.0256; **(C)** The triacylglycerol contents, mmol/L, in CON, AK1, AK2 and AK3 were 1.11 ± 0.13^b^, 1.40 ± 0.15^a^, 1.39 ± 0.08^a^, 1.40 ± 0.10^a^, respectively, *p* = 0.0245. ^a,b,c^ Values with different small letter superscripts above the figures mean a significant difference (*p* < 0.05).

The longissimus dorsi muscle inosinic acid, intramuscular fat, and triacylglycerol contents in groups treated with *Alpinia Katsumadai* extracts were higher (*p* < 0.05) than in CON, and the inosinic acid contents were higher (*p* < 0.05) in AK2 and AK3 than in AK1 (Figure 1).

### 3.3 Effects of *Alpinia Katsumadai* extracts on the longissimus dorsi muscle quality of Wuzhishan pigs

The b* and L* values at 45 min after slaughter of the longissimus dorsi muscle in groups treated with *Alpinia Katsumadai* extracts were significantly higher (*p* < 0.05) than in CON. The a* values at 45 min after slaughter were substantially higher (*p* < 0.05) than in CON, and they were higher (*p* < 0.05) in AK2 and AK3 than in AK1. The longissimus dorsi muscle drip loss in groups treated with *Alpinia Katsumadai* extracts was lower (*p* < 0.05) than in CON, and the shear force was lower (*p* < 0.05) in AK2 and AK3 than in CON and AK1 (Table 4).

**Table 4:** Effects of *Alpinia Katsumadai* extracts on the longissimus dorsi muscle quality of Wuzhishan pigs.

Items		CON	AK1	AK2	AK3	*p*-value
a*	45 min	3.51 ± 0.03^c^	3.67 ± 0.05^b^	3.79 ± 0.04^a^	3.80 ± 0.05^a^	0.0325
	24 h	4.01 ± 0.05	4.03 ± 0.07	3.99 ± 0.06	4.01 ± 0.09	0.0986
b*	45 min	4.56 ± 0.04^b^	4.96 ± 0.05^a^	4.94 ± 0.08^a^	4.95 ± 0.09^a^	0.0256
	24 h	4.00 ± 0.02	3.98 ± 0.04	3.99 ± 0.05	3.97 ± 0.04	0.0657
L*	45 min	50.85 ± 2.05^b^	55.06 ± 1.08^a^	55.21 ± 1.25^a^	55.31 ± 2.12^a^	0.0345
	24 h	44.21 ± 1.06	44.39 ± 2.01	44.31 ± 2.03	44.26 ± 2.09	0.0987
pH	45 min	6.25 ± 0.02	6.26 ± 0.03	6.24 ± 0.04	6.21 ± 0.03	0.0658
	24 h	5.47 ± 0.01	5.46 ± 0.02	5.48 ± 0.04	5.47 ± 0.02	0.0985
Shear force, *N*		63.25 ± 1.25^a^	63.05 ± 1.21^a^	58.02 ± 1.09^b^	57.86 ± 1.02^b^	0.0423
Drip loss, %		3.25 ± 0.02^a^	3.01 ± 0.04^b^	2.98 ± 0.06^b^	3.00 ± 0.05^b^	0.0238

^a,b,c^ Values with different small letter superscripts in the same column mean a significant difference (*p* < 0.05).

### 3.4 Effects of *Alpinia Katsumadai* extracts on longissimus dorsi muscle amino acid composition of Wuzhishan pigs

The longissimus dorsi muscle Gly, total amino acids, and essential amino acids contents in groups treated with *Alpinia Katsumadai* extracts were higher (*p* < 0.05) than in CON. The Ile and Lys contents were higher (*p* < 0.05) in AK1, AK2, and AK3 than in CON, and they were higher (*p* < 0.05) in AK2 and AK3 than in AK1. The Thr contents were higher (*p* < 0.05) in AK2 and AK3 than in CON and AK1 (Table 5).

**Table 5:** Effects of *Alpinia Katsumadai* extracts on longissimus dorsi muscle amino acid composition (g/kg) of Wuzhishan pigs.

Items	CON	AK1	AK2	AK3	*p*-value
Alanine, Ala	16.11 ± 0.06	16.13 ± 0.09	16.09 ± 0.10	16.11 ± 0.08	0.3957
Arginine, Arg	18.09 ± 0.12	18.12 ± 0.20	18.10 ± 0.11	18.11 ± 0.15	0.1256
Aspartic acid, Asp	15.23 ± 0.26	15.22 ± 0.24	15.24 ± 0.26	15.19 ± 0.19	0.2657
Glutamic acid, Glu	13.82 ± 0.19	13.81 ± 0.18	13.79 ± 0.28	13.81 ± 0.26	0.3287
Cysteine, Cys	3.56 ± 0.05	3.52 ± 0.06	3.53 ± 0.07	3.54 ± 0.08	0.5674
Glycine, Gly	11.32 ± 0.02^b^	11.41 ± 0.04^a^	11.42 ± 0.03^a^	11.40 ± 0.03^a^	0.0267
Histidine, His	10.56 ± 0.19	10.55 ± 0.18	10.54 ± 0.26	10.56 ± 0.27	0.0781
Isoleucine, Ile	8.57 ± 0.12^c^	9.02 ± 0.03^b^	9.11 ± 0.03^a^	9.12 ± 0.04^a^	0.0257
Leucine, Leu	17.15 ± 0.29	17.16 ± 0.31	17.14 ± 0.33	17.15 ± 0.34	0.0782
Lysine, Lys	18.01 ± 0.06^c^	18.16 ± 0.03^b^	18.25 ± 0.04^a^	18.25 ± 0.04^a^	0.0468
Methionine, Met	5.61 ± 0.12	5.63 ± 0.15	5.62 ± 0.16	5.63 ± 0.19	0.1564
Phenylalanine, Phe	10.12 ± 0.09	10.11 ± 0.08	10.13 ± 0.09	10.12 ± 0.07	0.2579
Proline, Pro	9.65 ± 0.15	9.67 ± 0.17	9.65 ± 0.19	9.66 ± 0.16	0.3564
Serine, Ser	11.72 ± 0.21	11.73 ± 0.22	11.72 ± 0.23	11.71 ± 0.21	0.4682
Threonine, Thr	10.61 ± 0.12^b^	10.68 ± 0.15^b^	11.25 ± 0.10^a^	11.23 ± 0.12^a^	0.0241
Tyrosine, Tyr	8.02 ± 0.20	7.98 ± 0.19	7.99 ± 0.19	8.01 ± 0.21	0.1591
Valine, Val	10.65 ± 2.12	13.59 ± 2.33	11.98 ± 2.84	11.89 ± 2.88	0.2846
Total amino acids	199 ± 1.12^b^	202 ± 1.15^a^	202 ± 1.20^a^	201 ± 1.21^a^	0.0316
Non-essential amino acids	89.43 ± 2.56	89.47 ± 2.24	89.43 ± 2.26	89.43 ± 2.64	0.3256
Essential amino acids	109 ± 1.29^b^	113 ± 1.20^a^	112 ± 1.21^a^	112 ± 1.22^a^	0.0216

Non-essential amino acids = Ala + Asp +Cys + Glu + Gly + Pro + Ser + Tyr. Essential amino acids = Lys + Met +Thr + Val + Ile + Leu + Phe + Arg + His. ^a,b,c^ Values with different small letter superscripts in the same column mean a significant difference (*p* < 0.05).

### 3.5 Effect of *Alpinia Katsumadai* extracts on longissimus dorsi muscle long-chain fatty acid proportion of Wuzhishan pigs

The longissimus dorsi muscle C14:0, C16:0, C18:1 n9t, C18:3 n3 proportions in groups treated with *Alpinia Katsumadai* extracts were significantly higher (*p* < 0.05) than in CON. The C18:2 n6c, C20:4 n6, and PUFA proportions were higher (*p* < 0.05) in AK1, AK2, and AK3 than in CON, and they were higher (*p* < 0.05) in AK2 and AK3 than in AK1. The C20:2 proportions were higher (*p* < 0.05) in AK2 and AK3 than in CON and AK1. The MUFA proportions in groups treated with *Alpinia Katsumadai* extracts were lower (*p* < 0.05) than in CON (Table 6).

**Table 6:** Effect of *Alpinia Katsumadai* extracts on longissimus dorsi muscle long-chain fatty acid proportion (%) of Wuzhishan pigs.

Items	CON	AK1	AK2	AK3	*p*-value
Myistic acid, C14:0	0.98 ± 0.01^b^	1.15 ± 0.01^a^	1.15 ± 0.02^a^	1.13 ± 0.02^a^	0.0325
Palmitic acid, C16:0	28.32 ± 0.12^b^	28.61 ± 0.10^a^	28.59 ± 0.12^a^	28.62 ± 0.13^a^	0.0459
Palmitoleic acid, C16:1	2.98 ± 0.32	3.25 ± 0.36	3.20 ± 0.28	3.21 ± 0.29	0.0512
Heptadeconic acid, C17:0	0.09 ± 0.01	0.09 ± 0.02	0.08 ± 0.01	0.08 ± 0.01	0.2631
Heptadecenoic acid, C17:1	0.12 ± 0.01	0.13 ± 0.03	0.11 ± 0.02	0.13 ± 0.01	0.3659
Stearic acid, C18:0	12.51 ± 0.56	12.50 ± 0.29	12.56 ± 0.30	12.49 ± 0.29	0.5876
Oleic acid (trans-9), C18:1 n9t	0.21 ± 0.01^b^	0.29 ± 0.01^a^	0.31 ± 0.03^a^	0.30 ± 0.03^a^	0.0256
Oleic acid (cis-9), C18:1 n9c	40.19 ± 1.25	39.06 ± 1.89	38.73 ± 1.58	38.67 ± 2.23	0.0562
Linoleic acid, C18:2 n6c	9.86 ± 0.02^c^	10.01 ± 0.01^b^	10.26 ± 0.03^a^	10.23 ± 0.03^a^	0.0269
γ-linolenic acid, C18:3 n6	0.09 ± 0.01	0.08 ± 0.01	0.08 ± 0.02	0.09 ± 0.01	0.5479
α-linolenic acid, C18:3 n3	0.31 ± 0.01^b^	0.36 ± 0.01^a^	0.36 ± 0.01^a^	0.37 ± 0.01^a^	0.0264
Arachidic acid, C20:0	0.32 ± 0.05	0.32 ± 0.02	0.32 ± 0.02	0.31 ± 0.01	0.2982
Arachidonic acid, C20:1	0.59 ± 0.02	0.59 ± 0.02	0.61 ± 0.03	0.60 ± 0.05	0.2598
Arachidonic acid, C20:2	0.31 ± 0.02^b^	0.31 ± 0.02^b^	0.36 ± 0.01^a^	0.36 ± 0.01^a^	0.0249
Arachidonic acid, C20:3 n6	0.28 ± 0.01	0.28 ± 0.02	0.28 ± 0.03	0.28 ± 0.01	0.0856
Arachidonic acid, C20:3 n3	0.13 ± 0.02	0.13 ± 0.02	0.15 ± 0.02	0.15 ± 0.02	0.1657
Arachidonic acid, C20:4 n6	1.59 ± 0.02^c^	1.71 ± 0.01^b^	1.81 ± 0.02^a^	1.83 ± 0.02^a^	0.0246
Docosanic acid, C22:0	1.12 ± 0.02	1.13 ± 0.02	1.04 ± 0.02	1.15 ± 0.03	0.5691
Saturated fatty acids, SFA	43.34 ± 3.56	43.80 ± 2.15	43.74 ± 2.48	43.78 ± 2.55	0.5983
Monounsaturated fatty acids, MUFA	44.09 ± 0.12^a^	43.32 ± 0.19^b^	42.96 ± 0.15^b^	42.91 ± 0.21^b^	0.0212
Polyunsaturated fatty acids, PUFA	12.57 ± 0.12^c^	12.88 ± 0.10^b^	13.30 ± 0.13^a^	13.31 ± 0.15^a^	0.0349

Saturated fatty acids = C14:0 + C16:0 + C17:0 + C18:0 + C20:0 + C22:0; monounsaturated fatty acids = C16:1 + C17:1 + C18:1 n9t + C18:1 n9c + C20:1; polyunsaturated fatty acids = C18:2 n6c + C18:3 n6 + C18:3 n3 + C20:2 + C20:3 n6 + C20:3 n3 + C20:4 n6. ^a,b,c^ Values with different small letter superscripts in the same column mean a significant difference (*p* < 0.05).

### 3.6 Effects of *Alpinia Katsumadai* extracts on expression of AMPK signaling regulatory pathway in longissimus dorsi muscle of Wuzhishan pigs

**Figure 2:**
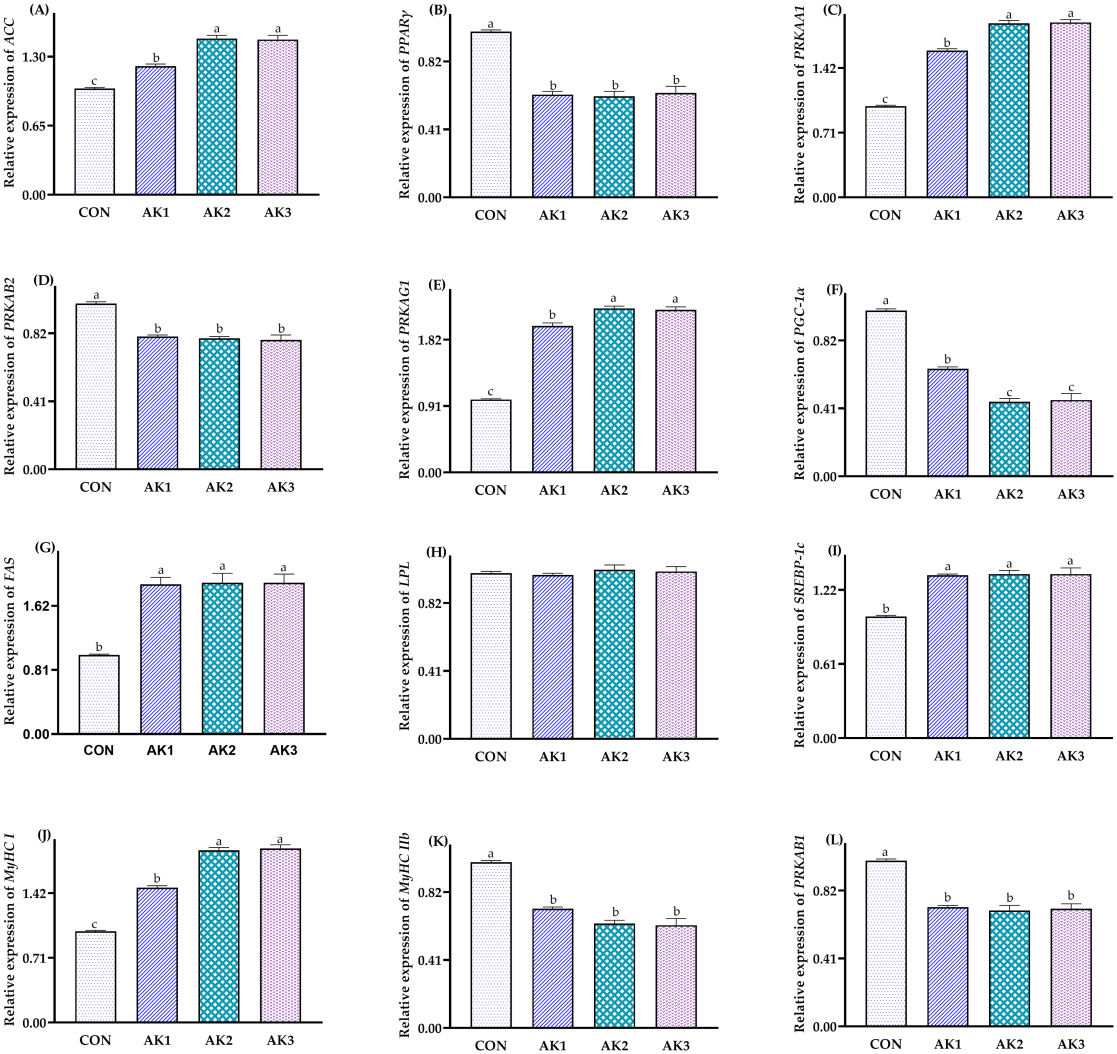
Effects of *Alpinia Katsumadai* extracts on expression of AMPK signal regulatory pathway in longissimus dorsi muscle of Wuzhishan pigs. **(A)** The data of relative expression levels of *ACC* (*acetyl-CoA carboxylase*) in group CON, AK1, AK2, AK3 were 1.00 ± 0.01^c^, 1.21 ± 0.02^b^, 1.47 ± 0.03^a^, 1.46 ± 0.04^a^, respectively, *p* = 0.0254; **(B)** The data of relative expression levels of *PPARγ* (*peroxisome proliferator-activated receptor gamma*) in group CON, AK1, AK2, AK3 were 1.00 ± 0.01^a^, 0.62 ± 0.02^b^, 0.61 ± 0.03^b^, 0.63 ± 0.04^b^, respectively, *p* = 0.0327; **(C)** The data of relative expression levels of *PRKAA1* (*protein kinase AMP-activated catalytic subunit alpha 1*) in group CON, AK1, AK2, AK3 were 1.00 ± 0.01^c^, 1.61 ± 0.02^b^, 1.91 ± 0.03^a^, 1.92 ± 0.03^a^, respectively, *p* = 0.0359; **(D)** The data of relative expression levels of *PRKAB2* (*protein kinase AMP-activated non-catalytic subunit beta 2*) in group CON, AK1, AK2, AK3 were 1.00 ± 0.01^a^, 0.80 ± 0.01^b^, 0.79 ± 0.01^b^, 0.78 ± 0.03^b^, respectively, *p* = 0.0368; **(E)** The data of relative expression levels of *PRKAG1* (*protein kinase AMP-activated non-catalytic subunit gamma 1*) in group CON, AK1, AK2, AK3 were 1.00 ± 0.01^c^, 2.01 ± 0.04^b^, 2.25 ± 0.03^a^, 2.23 ± 0.04^a^, respectively, *p* = 0.0269; **(F)** The data of relative expression levels of *PGC-1α* (*peroxisome proliferator-activated receptor-gamma coactivator 1-alpha*) in group CON, AK1, AK2, AK3 were 1.00 ± 0.01^a^, 0.65 ± 0.01^b^, 0.45 ± 0.02^c^, 0.46 ± 0.04^c^, respectively, *p* = 0.0364; **(G)** The data of relative expression levels of *FAS* (*fatty acid synthase*) in group CON, AK1, AK2, AK3 were 1.00 ± 0.01^b^, 1.89 ± 0.09^a^, 1.91 ± 0.12^a^, 1.91 ± 0.11^a^, respectively, *p* = 0.0251; **(H)** The data of relative expression levels of *LPL* (*lipoprotein lipase*) in group CON, AK1, AK2, AK3 were 1.00 ± 0.01, 0.99 ± 0.01, 1.02 ± 0.03, 1.01 ± 0.03, respectively, *p* = 0.0689; **(I)** The data of relative expression levels of *SREBP-1c* (*sterol regulatory element binding protein-1c*) in group CON, AK1, AK2, AK3 were 1.00 ± 0.01^b^, 1.34 ± 0.01^a^, 1.35 ± 0.03^a^, 1.35 ± 0.05^a^, respectively, *p* = 0.0356; **(J)** The data of relative expression levels of *MyHC I* (*myosin heavy chain I*) in group CON, AK1, AK2, AK3 were 1.00 ± 0.01^c^, 1.48 ± 0.02^b^, 1.89 ± 0.03^a^, 1.91 ± 0.04^a^, respectively, *p* = 0.0368; **(K)** The data of relative expression levels of *MyHC IIb* (*myosin heavy chain IIb*) in group CON, AK1, AK2, AK3 were 1.00 ± 0.01^a^, 0.72 ± 0.01^b^, 0.63 ± 0.02^b^, 0.62 ± 0.04^b^, respectively, *p* = 0.0462; **(L)** The data of relative expression levels of *PRKAB1* (*protein kinase AMP-activated non-catalytic subunit beta 1*) in group CON, AK1, AK2, AK3 were 1.00 ± 0.01^a^, 0.72 ± 0.01^b^, 0.70 ± 0.03^b^, 0.71 ± 0.03^b^, respectively, *p* = 0.0428. ^a,b,c^ Values with different small letter superscripts in the same column mean a significant difference (*p* < 0.05).

The relative expressions of *ACC*, *PRKAA1*, *PRKAG1*, and *MyHC I* in longissimus dorsi muscle in groups treated with *Alpinia Katsumadai* extracts were higher (*p* < 0.05) than in CON, and they were higher (*p* < 0.05) in AK2 and AK3 than in AK1. The relative expressions of *PRKAB1*, *PRKAB2*, *PPARγ*, and *MyHC IIb* in AK1, AK2, and AK3 were lower (*p* < 0.05) than in CON. The relative expressions of *PGC-1α* in groups treated with *Alpinia Katsumadai* extracts were lower (*p* < 0.05) than in CON, and they were lower (*p* < 0.05) in AK2 and AK3 than in AK1. The relative expressions of *FAS* and *SREBP-1c* were higher (*p* < 0.05) in AK1, AK2, and AK3 than in CON (Figure 2).

### 3.7 Effects of *Alpinia Katsumadai* extracts on expression of AMPK signaling regulatory pathway in liver of Wuzhishan pigs

**Figure 3:**
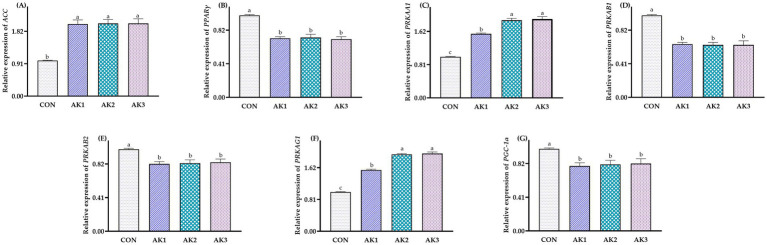
Effects of *Alpinia Katsumadai* extracts on expression of AMPK signal regulatory pathway in liver of Wuzhishan pigs. **(A)** The data of relative expression levels of *ACC* (*acetyl-CoA carboxylase*) in group CON, AK1, AK2, AK3 were 1.00 ± 0.01^b^, 2.02 ± 0.12^a^, 2.04 ± 0.11^a^, 2.04 ± 0.13^a^, respectively, *p* = 0.0454; **(B)** The data of relative expression levels of *PPARγ* (*peroxisome proliferator-activated receptor gamma*) in group CON, AK1, AK2, AK3 were 1.00 ± 0.01^a^, 0.72 ± 0.02^b^, 0.73 ± 0.04^b^, 0.71 ± 0.03^b^, respectively, *p* = 0.0273; **(C)** The data of relative expression levels of *PRKAA1* (*protein kinase AMP-activated catalytic subunit alpha 1*) in group CON, AK1, AK2, AK3 were 1.00 ± 0.01^c^, 1.56 ± 0.02^b^, 1.89 ± 0.05^a^,1.92 ± 0.06^a^, respectively, *p* = 0.0426; **(D)** The data of relative expression levels of *PRKAB1* (*protein kinase AMP-activated non-catalytic subunit beta 1*) in group CON, AK1, AK2, AK3 were 1.00 ± 0.01^a^, 0.65 ± 0.02^b^, 0.64 ± 0.03^b^, 0.64 ± 0.05^b^, respectively, *p* = 0.0284. **(E)** The data of relative expression levels of *PRKAB2* (*protein kinase AMP-activated non-catalytic subunit beta 2*) in group CON, AK1, AK2, AK3 were 1.00 ± 0.01^a^, 0.82 ± 0.03^b^, 0.83 ± 0.04^b^, 0.84 ± 0.04^b^, respectively, *p* = 0.0289; **(F)** The data of relative expression levels of *PRKAG1* (*protein kinase AMP-activated non-catalytic subunit gamma 1*) in group CON, AK1, AK2, AK3 were 1.00 ± 0.01^c^, 1.56 ± 0.02^b^, 1.96 ± 0.02^a^, 1.98 ± 0.04^a^, respectively, *p* = 0.0344; **(G)** The data of relative expression levels of *PGC-1α* (*peroxisome proliferator-activated receptor-gamma coactivator 1-alpha*) in group CON, AK1, AK2, AK3 were 1.00 ± 0.01^a^, 0.79 ± 0.04^b^, 0.81 ± 0.05^b^, 0.82 ± 0.06^b^, respectively, *p* = 0.0342. ^a,b,c^ Values with different small letter superscripts in the same column mean a significant difference (*p* < 0.05).

The relative expressions of *ACC*, *PRKAA1*, and *PRKAG1* in the liver in groups treated with *Alpinia Katsumadai* extracts were higher (*p* < 0.05) than in CON, and the relative expressions of *PRKAA1* and *PRKAG1* were higher (*p* < 0.05) in AK2 and AK3 than in AK1. The relative expressions of *PPARγ*, *PRKAB1*, *PRKAB2*, and *PGC-1α* in AK1, AK2, and AK3 were lower (*p* < 0.05) than in CON (Figure 3).

## 4 Discussion

### 4.1 Effects on production performance

In the livestock industry, enhancing animal production performance is of paramount significance (23, 24). Despite its importance, our review of the current literature indicates that research on the effects of *Alpinia Katsumadai* extracts on the production performance of poultry and livestock is limited. In our study, adding *Alpinia Katsumadai* extracts failed to significantly enhance the final body weight or carcass weight of Wuzhishan pigs. This outcome may be attributable to the specific growth stage of the pigs selected for the experiment. At this stage, the pigs’ digestive and metabolic systems are already highly developed, which likely limits the potential for exogenous additives to impact their body weight substantially. Another plausible explanation is that the dosage of *Alpinia Katsumadai* extracts used in this study may not have been sufficient to elicit a significant effect on the final body weight or carcass weight of Wuzhishan pigs. Additionally, other factors such as the specific growth stage of the pigs, the duration of the study, and the overall nutritional balance of the diet should be considered, as they could also influence the observed outcomes.

### 4.2 Effects on muscle composition

Inosinic acid is a key flavor compound in meat, imparting a pronounced umami taste that significantly enhances the overall flavor profile of meat products (25, 26). When paired with other flavor substances, such as guanosine, it can create a synergistic effect that amplifies the richness and deliciousness of meat (26, 27). Intramuscular fat is interspersed between and around myofiber groups and contains various flavor precursors (28, 29). It acts as a lubricant, reducing the friction between muscle fibers during chewing, thus enhancing the tenderness of meat (30, 31). Triglycerides represent the primary component of intramuscular fat and serve as the main form of energy storage in meat. During cooking, these molecules can be hydrolyzed into glycerol and fatty acids. This breakdown process facilitates energy release while producing a range of flavor compounds that contribute to the overall taste of the meat (32–34). The increased inosinic acid, intramuscular fat, and triacylglycerol in the longissimus dorsi muscle of pigs treated with *Alpinia Katsumadai* extracts suggest potential improvements in meat quality. Inosinic acid is a key contributor to the flavor profile of meat, and its higher levels in the treated groups, particularly in AK2 and AK3, indicate an enhancement in taste (26). Although the exact mechanism by which *Alpinia Katsumadai* extracts increase inosinic acid levels is not fully elucidated in this study, it is plausible that the bioactive compounds in the extracts may influence the activity of enzymes involved in its synthesis pathway, such as AMP deaminase, thereby promoting the production of inosinic acid and ultimately improving the flavor of the meat. In future research, we will investigate the specific interactions between *Alpinia Katsumadai* extracts and the enzymes responsible for inosinic acid synthesis to clarify this mechanism further. In this study, the finding aligns with previous studies exploring plant extracts’ role in modulating meat flavor compounds. For instance, Bertocci (35), Martínez (36), and Onopiuk (37) reported that certain plant-derived compounds can stimulate the production of flavor-enhancing substances in meat, thereby improving its palatability. Elevating intramuscular fat content is also desirable, as it is associated with enhanced juiciness and tenderness of the meat (29, 30). This is consistent with the general understanding in meat science that a higher contents of intramuscular fat, leads to better eating quality (38, 39). The results from this study, showing a significant increase in intramuscular fat in the treated groups compared to the control, support the potential of *Alpinia Katsumadai* extracts as a natural means to enhance this quality attribute.

### 4.3 Effects on muscle quality

Meat color is a significant criterion consumers use to evaluate meat quality and acceptability, as demonstrated in various consumer studies (40, 41). A visually appealing color can significantly enhance the attractiveness of meat products, influencing consumer purchasing decisions (42). The significant improvements in the longissimus dorsi muscle L* in the treated groups highlight the potential of *Alpinia Katsumadai* extracts to enhance the visual appeal and perceived quality of the meat. The higher a* in the treated groups, especially in AK2 and AK3, suggests a more desirable red color, often associated with freshness and quality in consumer perception (41). This is supported by Altmann’s study, which discusses the importance of meat color in consumer acceptance (42). Lipid oxidation is critical to meat color deterioration (43). The antioxidant components in essential oils can effectively remove free radicals in muscles and inhibit peroxidation reactions, thereby maintaining the fresh color of the meat and making the meat color more ruddy and bright (44). Plant essential oils can impart unique and desirable flavors to meat products, enhancing their sensory appeal (45, 46), which may be because the *Alpinia Katsumadai* extracts improved the color of the longissimus dorsi muscle in Wuzhishan pigs through its essential oil components, and the improvement was manifested in the form of L*, and a*.

From a sensory perspective, meat with low drip loss tends to have better texture and flavor (47, 48). Shear force is used to quantify the tenderness of meat, with lower shear force values indicating more tender meat (49, 50). The reduction in drip loss and shear force in the treated groups suggests an improvement in the water-holding capacity and tenderness of the meat. These findings are crucial as they directly relate to the economic value and consumer satisfaction of the meat product. Lower drip loss means less loss of valuable meat juice during storage and cooking, while a lower shear force implies that the meat is easier to cut and chew (48, 50). Cebulska (51) and Xu (52) explored the effects of supplement plant extracts on meat tenderness and water-holding capacity, emphasizing the importance of these plant extracts in meat quality assessment. In this study, the improvement of drip loss and shear force in the longissimus dorsi muscle of Wuzhishan pigs may be due to the antioxidant components in the essential oil of *Alpinia Katsumadai* extracts, which can effectively remove free radicals in the muscle and inhibit the occurrence of oxidation reactions (53). This protects the structural integrity of muscle proteins, reduces protein cross-linking and denaturation caused by oxidation, and helps maintain the meat’s tenderness (53, 54).

### 4.4 Effects on amino acid composition and long-chain fatty acid proportion

Amino acids and long-chain fatty acids are crucial in determining meat nutrition and developing flavor (55, 56). The increase in the contents of glycine, total amino acids, and essential amino acids in the longissimus dorsi muscle of pigs treated with *Alpinia Katsumadai* extracts suggests potential improvements in the nutritional value of meat products. Amino acids are fundamental to the nutritional value of meat. The specific increases in isoleucine, lysine, and threonine, essential amino acids, are noteworthy. These amino acids play critical roles in various physiological functions, including muscle growth, repair, and immune function (57, 58). The findings from this study suggested that *Alpinia Katsumadai* extracts could enrich the nutritional content of meat, which implied the potential of *Alpinia Katsumadai* extracts to modulate the amino acid composition of animal products. This may be because the terpenoid and phenolic compounds in the essential oil of *Alpinia Katsumadai* extracts could promote the synthesis of amino acids through specific enzymes or signaling pathways and also interact with amino acid transport proteins to regulate the transport of amino acids across cell membranes, thereby improving the content of specific amino acid components in the meat (59, 60).

The alterations in the long-chain fatty acid profile of the longissimus dorsi muscle in the treated groups, with an increase in C14:0, C16:0, C18:1 n9t, C18:3 n3, and PUFA, and a decrease in MUFA, had significant implications for the health benefits of the meat. In particular, polyunsaturated fatty acids (PUFAs) are known for their health-promoting properties, such as reducing the risk of cardiovascular diseases. The upregulation of *ACC* (mentioned below) in the treated groups may seem counterintuitive given its typical role in promoting the synthesis of saturated fatty acids (SFAs). However, it is essential to note that *ACC* is a key enzyme in fatty acid synthesis, and its upregulation could potentially support overall fatty acid production. Despite the upregulation of ACC, the observed increase in PUFA proportions may be attributed to the complex interplay of various metabolic pathways and regulatory mechanisms influenced by *Alpinia Katsumadai* extracts. These extracts may modulate the activity of other enzymes or regulatory proteins involved in fatty acid desaturation and elongation, thereby promoting the synthesis of PUFAs. Additionally, the extracts could influence the expression or activity of *peroxisome proliferator-activated receptors* (*PPARs*), which are known to regulate fatty acid metabolism and can promote the synthesis of PUFAs. In future research, we will focus on elucidating the specific mechanisms by which *Alpinia Katsumadai* extracts influence the balance between SFA and PUFA synthesis, including the potential involvement of ACC and other key regulatory factors.

Hao (61), Grela (62), and Hervé (63) have examined the impact of dietary plant extract interventions on the fatty acid composition of meat and their potential health benefits. Their findings align closely with the results of the present study. The results from this study indicated that *Alpinia Katsumadai* extracts could be used to manipulate the fatty acid composition of meat in a way that enhances its health value. This may be because the essential oil of *Alpinia Katsumadai* extracts activates fatty acid synthase, promoting the synthesis of fatty acids and increasing the content of long-chain fatty acids in the meat (64, 65).

### 4.5 Effects on regulation of AMPK signaling pathway

The *ACC* is a class of genes that encode acetyl-CoA carboxylase, which plays a role in fatty acid synthesis (66). Its expression is regulated by various factors, including hormones and nutritional status (67). *PPARγ* plays a crucial role in the differentiation of adipocytes. It can regulate fatty acid synthesis, storage, and breakdown in adipose tissue, as well as carbohydrate metabolism and lipid metabolism in the liver (68). The *PRKAA1*, *PRKAB1*, *PRKAB2*, and *PRKAG1* encode the catalytic subunit of AMP-activated protein kinase (AMPK) (69). The AMPK regulates metabolic processes such as fatty acid, glycogen, and protein synthesis by phosphorylating multiple key metabolic enzymes (70). *PGC-1 α* plays a crucial role in metabolic processes such as fatty acid oxidation, glycogen synthesis, and protein synthesis, thereby regulating cellular energy metabolism (71). The *FAS* encodes fatty acid synthase, a multi-enzyme complex (72). Its primary function is to catalyze the synthesis of long-chain saturated fatty acids from acetyl-CoA and malonyl-CoA in the presence of NADPH (72). The *LPL* encodes lipoprotein lipase, a multifunctional enzyme (73). Its primary function is to catalyze the hydrolysis of triglycerides in chylomicrons and very low-density lipoproteins, producing fatty acids and monoglycerides used by tissues (74). *SREBP-1c* is a significant regulatory factor in fatty acid synthesis, capable of activating multiple enzyme genes involved in fatty acid synthesis, such as acetyl-CoA carboxylase and fatty acid synthase, thereby promoting *de novo* fatty acid synthesis (75, 76). The AMPK signaling pathway regulates the expression and activity of myosin heavy chain through multiple mechanisms, which can affect muscle fiber type transition and cell migration capabilities (77). In muscle tissue, the AMPK facilitates the conversion of fast-twitch muscle fibers to slow-twitch muscle fibers by upregulating the expression of *PGC-1α*, increasing the expression of myosin heavy chain subtypes associated with slow-twitch fibers (78). Additionally, the AMPK modulates mitochondrial dynamics, maintaining an imbalance in energy levels that further enhances its signaling pathway and the activity of *Myosin Heavy Chain II* (77).

The differential expression of genes associated with the AMPK signaling pathway in the longissimus dorsi muscle of treated pigs offers valuable mechanistic insights into the potential modes of action of *Alpinia Katsumadai* extracts. The upregulation of *ACC*, *PRKAA1*, *PRKAG1*, and *MyHC I*, and the downregulation of *PRKAB1*, *PRKAB2*, *PPARγ*, and *MyHC IIb*, suggest a shift in muscle metabolism and fiber type composition. The AMPK pathway is a central regulator of cellular energy homeostasis, and its activation can lead to increased fatty acid oxidation and glucose uptake, among other metabolic adjustments. The findings from this study suggest that *Alpinia Katsumadai* extracts may modulate muscle metabolism via the AMPK pathway, thereby potentially contributing to the observed improvements in meat composition and quality. This conclusion is further supported by the work of Wang (79) and Guo (80), who have detailed the role of AMPK in muscle metabolism and elucidated how its modulation can influence meat quality traits. However, we must note some differences in gene expression patterns between our study and theirs. For instance, while both studies observed the upregulation of specific AMPK-related genes such as *ACC* and *PRKAA1*, their research did not explicitly discuss the downregulation of *PPARγ*. This discrepancy may be due to differences in the particular plant extracts, animal models, or experimental conditions.

The observed alterations in the expression of AMPK-related genes in the livers of treated pigs further elucidate the systemic impact of *Alpinia Katsumadai* extracts, expanding our understanding of their multifaceted physiological effects. The upregulation of *ACC*, *PRKAA1*, and *PRKAG1*, and the downregulation of *PPARγ*, *PRKAB1*, *PRKAB2*, and *PGC-1α*, suggest that the extracts may also influence whole-body metabolism by acting on the liver, which is a key organ for metabolic regulation (81). The liver is a central hub for lipid and glucose metabolism, and modulating the AMPK pathway within this organ can have profound and far-reaching effects on an animal’s overall metabolic profile. Enhancing meat quality through targeted modulation of metabolic pathways represents a novel and promising approach. This concept is supported by Zhang’s study (82), which elucidated the intricate interplay between liver metabolism and meat quality in animals.

### 4.6 Evaluation of the optimal dosage

Based on the results for muscle composition, muscle quality, amino acid and long-chain fatty acid profiles, as well as the relative expression levels of genes in the AMPK signaling pathway, *Alpinia Katsumadai* extracts exerted significant beneficial effects on multiple parameters of the longissimus dorsi muscle in Wuzhishan pigs within the dosage range of 400 to 800 mg/kg. Notably, these improvements tended to plateau at 600 mg/kg and 800 mg/kg, with no further significant enhancement observed at the higher dose. In some cases, specific indicators even showed slight declines or stabilized at elevated dosages. This pattern suggests a dosage threshold, beyond which the positive effects of *Alpinia Katsumadai* extracts on muscle quality, composition, and gene expression may reach a relatively steady equilibrium. Further increases in dosage may offer limited additional benefits and could potentially trigger negative feedback mechanisms aimed at maintaining metabolic homeostasis (83). At higher dosages, although the extract continued to exert significant promotive effects on specific parameters, the expression of some genes began to plateau or slightly decline, and the levels of specific metabolites stabilized or showed modest reductions. These observations suggest that the organism may activate negative feedback regulatory mechanisms to prevent excessive metabolic fluctuations and to preserve physiological function and metabolic homeostasis. This regulatory response likely involves the integration of multiple signaling pathways that govern energy balance and nutrient metabolism. In particular, pathways responsible for sensing intracellular energy status and nutrient availability may adjust gene expression and enzyme activity to maintain metabolic equilibrium. Under sustained high-dose exposure to *Alpinia Katsumadai* extracts, such mechanisms may constrain further metabolic stimulation, thereby plateauing the observed beneficial effects.

In summary, *Alpinia Katsumadai* extracts demonstrated significant improvements across multiple parameters of the longissimus dorsi muscle in Wuzhishan pigs within the dosage range of 400 to 800 mg/kg. The 600 mg/kg dosage yielded the most favorable overall effects among these. Further increasing the dosage to 800 mg/kg resulted in stabilizing the improvements, which may be attributed to a threshold effect and the activation of potential negative feedback mechanisms at higher doses. In future research, we will focus on elucidating the underlying mechanisms of *Alpinia Katsumadai* extracts and the specific metabolic responses and regulatory pathways engaged at different dosage levels. These insights will provide a more scientific basis for optimizing its application in swine production.

## 5 Conclusion

This study examined the impact of dietary *Alpinia Katsumadai* extracts on meat quality in Wuzhishan pigs. The results revealed significant enhancements in muscle composition, with notable increases in inosinic acid, intramuscular fat, and triacylglycerol content. Meat quality was significantly improved, as indicated by elevated color values (b*), reduced drip loss, and decreased shear force, collectively suggesting enhanced tenderness and water-holding capacity. The amino acid profile was also enriched, with higher levels of essential amino acids such as glycine, isoleucine, and lysine. Additionally, the fatty acid composition shifted favorably, with increased proportions of beneficial fatty acids like C14:0, C16:0, C18:3 n3, and PUFA, and a decrease in MUFA proportions. Importantly, these changes were accompanied by significant alterations in the expression of genes involved in the AMPK signaling pathway, suggesting a potential mechanism through which *Alpinia Katsumadai* extracts modulate meat quality. The optimal dosage identified in this study was 600 mg/kg. These findings underscore the potential of *Alpinia Katsumadai* extracts as a natural additive to improve meat quality and nutritional value in pig production.

## Data Availability

The raw data supporting the conclusions of this article will be made available by the authors, without undue reservation.
